# Twenty years of efforts to reduce stunting in Ecuador: how to accelerate progress and confront the challenges?

**DOI:** 10.1016/j.lana.2026.101386

**Published:** 2026-02-09

**Authors:** Jairo Rivera, Estefani Jarrín, Ximena Garzon-Villalba, José Ruales

**Affiliations:** a Universidad Andina Simón Bolívar, Toledo N22-80, Quito, Ecuador; b Pontificia Universidad Católica del Ecuador, Av. 12 de Octubre 1076, Quito, Ecuador; c Universidad San Francisco de Quito, Av. Diego de Robles & Vía Interoceánica, Quito, Ecuador; d Universidad Internacional SEK, Campus Miguel de Cervantes Calle Alberto Einstein S/N y 5ta. Transversal, Quito, Ecuador

Stunting, which is defined as impaired growth and development in children, is intrinsically linked to social determinants of health. Eliminating stunting is a matter of social justice and human rights. Global nutritional targets emphasize protecting the first 1000 days of life.^1^ The determinants of stunting arise from immediate, underlying, and structural factors.^2^ The consequences of stunting extend across the life course, with significant effects on cognitive development, productivity, and overall well-being.^3^

Globally, over the past two decades, the prevalence of stunting among children under five years old has decreased from 31.6% in 2004 to 23.2% in 2024.^4^ In Latin America and the Caribbean (LAC), stunting fell from 16.2% in 2004 to 12.4% in 2024, below the global average. However, stark inequalities persist: while some countries, such as Chile, have achieved prevalence rates below 2%, others, such as Guatemala, report rates exceeding 40%.^4^

Ecuador is among the countries with the highest stunting prevalence in LAC.^5^^,^^6^ The period 2004–2014 has reduced the prevalence of stunting among children under five, but has increased it among children under two years.^7^ Fortunately, the reduction in stunting for both age groups has accelerated in the period 2014–2024. Stunting among children under five declined from 28.9% in 2004 to 17.5% in 2024, with an average annual reduction of 0.6 percentage points. Among children under two, stunting decreased from 21.3% in 2004 to 19.3% in 2024, despite peaking at 24.8% in 2014.

The acceleration on the rate of reduction over the last five years coincides with the creation of the *National Strategy Ecuador Grows Without Stunting*^8^ and the establishment of the Technical Secretariat Ecuador Grows Without Stunting to coordinate public policy, implement a prioritized maternal and child package of interventions, develop a universal interconnected nominal monitoring system, carry out a specialized survey, and to create an advisory council composed of civil society organizations. These efforts have strengthened national recognition of stunting in both public and political spheres and increased societal awareness. This progress is reflected in the recent approval of a package of Laws and the renewal of the Intersectoral Strategic Plan for the Prevention and Reduction of stunting 2025–2030.^9^

Despite these notable achievements, significant structural challenges, such as drinking water and sewage coverage,^10^ remain, and current challenges have appeared, such as the creation of a new Ministry of Human Development, which assumes some of the programs against children stunting following the elimination of the Technical Secretariat Ecuador Grows Without Stunting.^11^ Although many interventions have been implemented, such as the conditional cash transfer “Bono 1000 Días” their impact on nutrition has not been as substantial as expected.^12^ One possible explanation relates to gaps in scientific research that limit the ability to identify causal pathways specific to Ecuador's context and hinder robust policy evaluation and impact analysis.

To address this gap, the “Scientific Research in Maternal and Child Health and Nutrition Grant” has been launched by the Fundación Ecuador Crece Contigo, which aims to promote academic research.^13^

Through this initiative, Fundación Ecuador Crece Contigo seeks to strengthening rigorous analysis, and to promote prevention and control programs tailored to the Ecuadorian context, essential to accelerate progress and to reduce the social and economic impacts of this public health challenge.

## Contributors

Conceptualization: JRivera, EJarrín.

Data curation: JRivera.

Formal analysis: JRivera, EJarrín, XGarzón-Villalba, JRuales.

Investigation: JRivera, EJarrín, XGarzón-Villalba, JRuales.

Methodology: JRivera, EJarrín, XGarzón-Villalba, JRuales.

Supervision: EJarrín, XGarzón-Villalba, JRuales.

Validation: JRivera, EJarrín, XGarzón-Villalba, JRuales.

Visualization: JRivera, EJarrín, XGarzón-Villalba, JRuales.

Writing—original draft: JRivera, EJarrín.

Writing—review & editing: JRivera, EJarrín, XGarzón-Villalba, JRuales.

Submission: JRivera, EJarrín, XGarzón-Villalba, JRuales.

## Declaration of interests

Ximena Garzón, José Ruales, Estefani Jarrín are members of the Board of Directors of Fundación Ecuador Crece Contigo. Jairo Rivera is a member of the Consulting Team of Fundación Ecuador Crece Contigo.
